# Through every lens: assessing the impact of chemical modifications on antibody-conjugates using in vivo imaging

**DOI:** 10.1038/s44303-025-00109-8

**Published:** 2025-10-28

**Authors:** Veera V. Shivaji R. Edupuganti, Freddy E. Escorcia, Martin J. Schnermann

**Affiliations:** 1 Chemical Biology Laboratory, Center for Cancer Research, National Cancer Institute, Frederick, MD USA; 2 Molecular Imaging Branch, Center for Cancer Research, National Cancer Institute, Bethesda, MD USA

**Keywords:** Cancer, Chemical biology, Chemistry, Nanoscience and technology

## Abstract

Chemical modification of monoclonal antibodies (mAbs) and their fragments gives rise to imaging probes and targeted therapies. Depending on the isotope used, radiolabeled mAbs enable positron emission tomography (PET) and single photon emission computed tomography (SPECT) imaging and can also be applied as cytotoxic therapies. Fluorescent mAb conjugates are used for a range of preclinical applications with clinical utility for intraoperative visualization of tumors. Antibody-drug conjugates (ADCs) enhance the therapeutic efficacy of mAbs and are the topic of extensive clinical development. In all these cases, chemical modifications can significantly affect mAb tumor targeting and clearance. Whole-body imaging techniques provide crucial insights into the in vivo consequences of these changes by directly tracking antibody conjugate distribution and clearance. This review examines in vivo imaging studies that compare “parental” and “modified” mAbs imaged under identical conditions to assess the effects of the cargo itself (e.g. fluorophore, chelator, drug), as well as the chemical conjugation methods. Additionally, we also describe studies that evaluate alternative strategies, including pretargeting, Fc modifications and pre- or co-dosing strategies that seek to tune the biodistribution of a given conjugate. Overall, we highlight the critical role of imaging in characterizing the in vivo performance of mAb conjugates, underscoring how these insights can inform both therapeutic efficacy and toxicity, and enable clinical translation.

## Introduction

Monoclonal antibodies (mAbs) have become essential therapeutics in many clinical settings. Functionalization of native mAbs with small molecules confers additional applications in diagnostic imaging and therapeutic settings^1^. Depending on the isotope, radionuclides coupled to mAbs can facilitate PET and SPECT imaging for diagnostic staging of patients with cancer and antigen quantitation, or serve as cytotoxic therapeutic agents, a strategy often referred to by the portmanteau “radiotheranostics”^2–4^. Fluorophore-mAb conjugates find broad preclinical use for a range of in vitro and in vivo studies and facilitate intraoperative imaging to enable real-time definition of tumor margins^5,6^. Finally, antibody-drug conjugates (ADCs), comprised of small molecule drug payloads coupled to mAbs, have emerged as a successful, rapidly expanding modality to treat patients with a spectrum of cancer types^7^.

Understanding the pharmacokinetics (PK) of antibodies and their conjugates is crucial for optimizing their desired function^8^. Critically, chemical modification can significantly influence the PK properties of antibodies by altering their absorption, distribution, metabolism, and elimination (ADME). In many cases, chemical modification decreases tumor targeting while increasing clearance rates compared to the parental mAb. These PK changes occur due to a complex mixture of factors, sometimes involving changes in the binding affinity of the antibody to its target, but, more often, are due to changes in interactions with various clearance-related processes, particularly in the reticuloendothelial system.

**Fig. 1:**
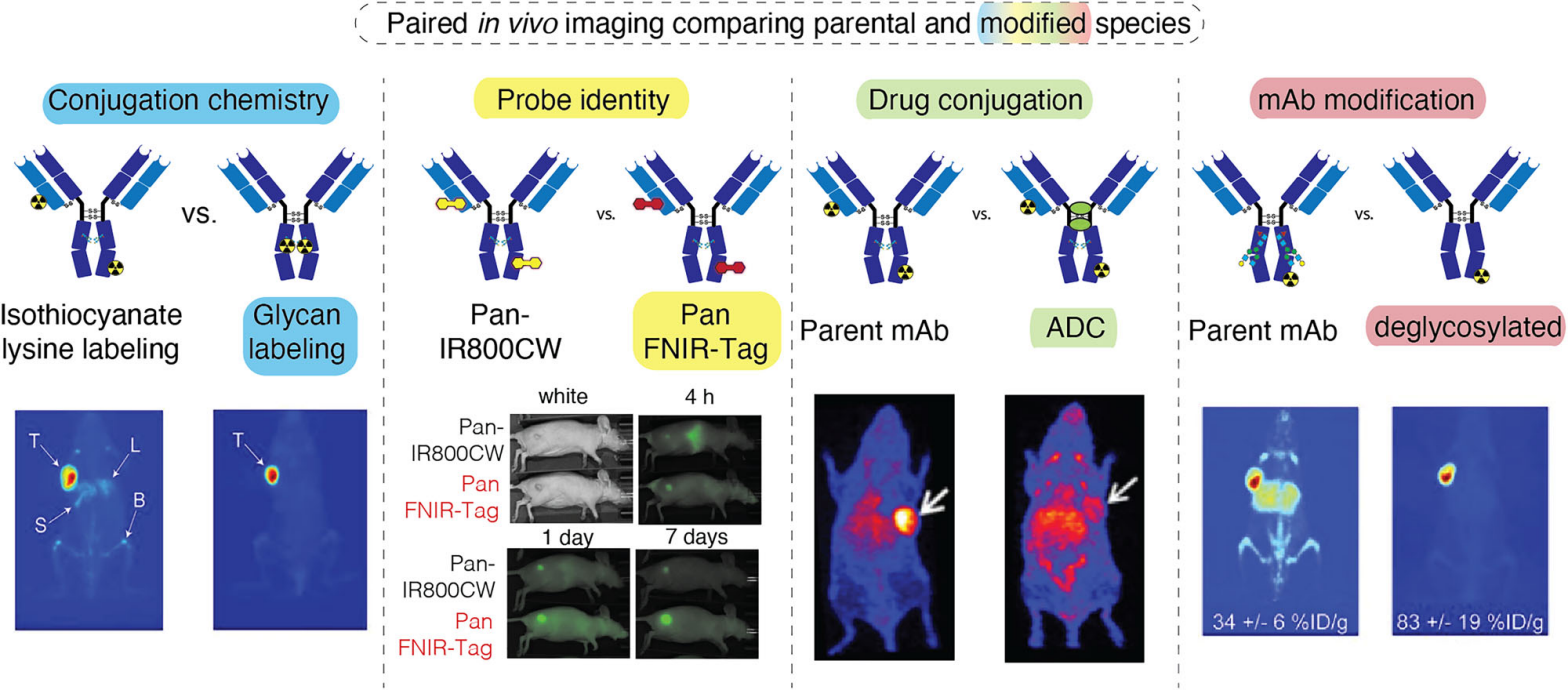
Representative comparisons. Head-to-head comparisons with sample images of studies that use in vivo imaging to assess the impact of altered conjugation chemistry, probe identity, drug conjugation, and mAb modification. Figure adapted from refs. ^30,63,71^, and ^78^.

Several methods can assess the impact of chemical modification on antibody targeting. Blood pool measurements using enzyme-linked immunosorbent assay (ELISA) and mass spectrometry remain the most common and are essential for the definition of various PK parameters (e.g. blood half-life)^9^. Complementing these methods, optical- and radionuclide-based whole body imaging “lenses” can determine the location of the administered agent longitudinally. These methods are useful to define not only tumor localization of a given agent, but also uptake in clearance organs (i.e., liver and kidney) and non-target tissues. While initially limited to murine pre-clinical models, there are now many examples using optical- and radionuclide-labeled mAbs in clinical settings. The goal of this review is to examine the unique role that whole body in vivo imaging studies can play in assessing how modifications to mAb and mAb fragments affect biodistribution. We have sought to identify recent, representative studies where both a “parental” (e.g. unmodified) and “modified” antibody or antibody fragment containing the cargo of interest (e.g. radionuclide, fluorophore, drug) species were imaged under identical conditions (see Fig. 1 for examples). Such paired studies allow the quantitative assessment of the impact of a given modification. This review is organized around the data presented in Table 1. We first examine the role of the bioconjugation chemistry linking the imaging probe and mAb. We then describe examples where the impact of the identity of the cargo has been carefully examined. Finally, we described strategies being explored to improve tumor binding and minimize off-target binding, including changes to the mAb itself, particularly in the Fc domain, in vivo pretargeting approaches, and altered dosing regimens. We note that various nanomaterial- and peptide or small molecule-based agents also have great promise but are beyond the scope of this review^4,10–12^. Overall, we hope to illustrate the potential of in vivo imaging to provide design insights that improve the targeting of mAb conjugates—a complex problem with significant clinical implications.

**Table 1:** Select paired in vivo imaging studies with details about the study, the key modification and its impact

	Imaging modality	Target	Cell line	Protein type (name)	Parent agent (labeling)	Modification (labeling)	Impact of modification relative to parent (time)	Ref.
Labeling chemistry	PET (^89^Zr)	HER2	SW1222	mAb (huA33)	DFO (Maleimide)	DFO (PODS)	1.8x ↑ TLR, 1.6 ↑ TKR	^26^
	PET (^89^Zr)	HER2	BT-474	mAb (Pertuzumab)	DFO (NHS)	DFO (glycoconnect)	2.4x ↑ Tumor and 2.2x ↓ Liver (120 h)	^30^
	PET (^89^Zr)	HER2	Clinical	mAb (Pertuzumab)	DFO (NHS)	DFO (glycoconnect)	Improved lesion detection and higher tracer avidity	^28^
	PET (^89^Zr)	GPC3	A431-GPC3+	VHH (HN3)	DFO (NHS)	DFO (Sortase)	2.3x ↑ TLR (1 h)	^32^
	Fluorescence	HER2	JIMT-1	VHH (2Rs15d)	IRDye800CW (NHS)	IRDye800CW (Maleimide C-terminal cysteine)	4.1x ↑ TMR (24 h)	^39^
	Fluorescence	CD276	JIMT-1	mAb (m276SL)	Cy7 (Maleimide interchain)	Cy7 (S239C Maleimide)	4.2x ↑ TBR (72 h), 3.1x ↓ liver (4 h)	^40^
	Fluorescence	EGFR	MDA-MB-468	mAb (Panitumumab)	Disulfo-FNIR (NHS)	Disulfo-FNIR (PFP)	1.9x ↑ TBR (168 h)	^42^
	PET (^89^Zr)	HER2	BT-474	mAb (Pertuzumab)	DFO (Maleimide interchain & NHS)	DFO DBCO alkyne (PFP azide)	no statistically significant differences	^43^
Imaging agent	PET (^89^Zr)	HER2	N87	mAb (Trastuzumab)	DFO (NCS)	DFO* (NHS)	1.1x ↑ Tumor, 5.9x ↓ Bone (144 h)	^51^
	PET (^89^Zr)	CD11b	J774A.1	mAb (αCD11b)	DFO (NCS)	Lumi 804 (NHS)	12.6x ↓ Bone, 5.2x ↓ Kidney and 24x ↓ Intestinal (216 h)	^52^
	PET (^89^Zr)	EGFR	A431	VHH (7D12)	RESCA Al-F (Sortase)	Fpy (Sortase PFP)	~4x ↑ TBR (3 h), ~127x ↑ TKR (3 h)	^53^
	Fluorescence	CEA	LS174T	mAb (M5A)	IRDye800CW (Maleimide interchain)	Sidewinder (Bromoacetamide interchain)	1.8x ↑ TBR (48 h)	^56^
	Fluorescence	EGFR	MDA-MB-468	mAb (Panitumumab)	IRDye800CW (NHS)	FNIR-Tag (NHS)	~3.9x ↑ TBR (7 d), ~4.6x ↓ LBR (4 h)	^63^
	Fluorescence	EGFR	JIMT-1	mAb (Panitumumab)	IRDye800CW (NHS)	FNIR-Tag-766 (NHS)	19x ↑ Tumor (72 h)	^61^
	Fluorescence	EGFR	JIMT-1	VHH (7D12)	IRDye800CW (NHS)	FNIR-Tag-766 (NHS)	12.5x ↑ TLR (1 h)	^61^
Antibody-drug conjugate	Fluorescence	CD38	MM.1S	mAb (Daratumumab)	IRDye800CW (NHS)	IR800CW (NHS) & DM1 (NHS)	1.2x ↓ Tumor (8 d), 1.8x ↑ Liver (9 d)	^69^
	Fluorescence	5T4	H1975	mAb (5T4)	VT680 (NHS)	VT680 (NHS) & mcMMAF (Maleimide interchain)	1.5x ↓Tumor, (10 d)	^70^
	PET (^89^Zr)	EphA2	PC3	mAb (1C1)	DFO (NCS)	DFO (SCN) & AZ13599185 (S239C)	~1.9x ↓ Tumor, ~1.4x ↓ Liver (72 h)	^71^
	SPECT (^111^In)	CD174	Clinical	mAb (G193)	CHX-A”-DTPA (NCS)	CHX-A”-DTPA (NCS) & calicheamicin (Maleimide interchain)	19.3x ↓ Tumor, 4.5x ↑ Liver (24 h)	^72^
Miscellaneous	SPECT (^111^In)	HER2	KPL-4	mAb (7C2)	DOTA (Maleimide)	YTE-KF mAb mutations	1.7x ↓ Tumor, 4.2 ↓ blood (48 h)	^73^
	PET (^89^Zr)	HER2	BT-474	mAb (Trastuzumab)	DFO (NCS)	DFO (NCS) & PNGaseF treatment	2.1x ↑ Tumor, ~3x ↓ spleen (120 h)	^78^
	SPECT (^111^In)	HER2	KPL-4	mAb (7C2)	DOTA (Maleimide)	Maleimide TCO + DOTA tetrazine @ 24 h	4.7x ↓ Tumor (48 h)	^73^
	SPECT (^111^In)	TENB2	LuCaP 77	mAb (anti-TENB2)	DOTA (Maleimide)	Predosing with anti-TENB2 (1 mg/kg)	no Δ tumor, 2.1x ↓ liver (72 h)	^84^

For each example, we attempted to identify the most significant difference obtained through the indicated modification. The cell line indicated was the one used in the relavent in vivo study, even if other cell lines were also tested. When precise values are included, those are used with 2 significant figures. When estimating values off bar graphs, an approximation symbol (~) is included. When a single organ or tumor is listed, the comparison is the ratio of modified/ parental species uptake (%ID/g when possible) and the fold change up or down. A similar approach is taken for the indication changes in ratio ratio, using the published ratio values.

*TBR* Tumor-to-Background Ratio, *TLR* Tumor-to-Liver Ratio, *TMR* Tumor-to-Muscle Ratio, *TKR* Tumor-to-Kidney Ratio, *KBR* Kidney-to-Background Ratio, *DFO* Deferoxamine, *ITC* Isothiocyanate, *mAb* Full Monoclonal Antibody, *NHS* N-hydroxy succinimide, *CHX-A DTPA* 2-(p-isothiocyanatobenzyl)-cyclohexyl-diethylenetriaminepentaacetic acid, *VHH* Variable heavy domain antibody fragments, *HER2* human epidermal growth factor receptor 2, *EGFR* Epidermal growth factor receptor, *5T4* trophoblast glycoprotein, *CEA* Carcinoembryonic antigen, *TENB2* tomoregulin, *EphA2* Eph Receptor A2, *DBCO* dibenzocyclooctyne, *PFP* pentafluorophenyl, *FPy* fluoropyridine, *PNGaseF* Peptide:N-glycosidase F.

## Antibody labeling chemistry

**Fig. 2:**
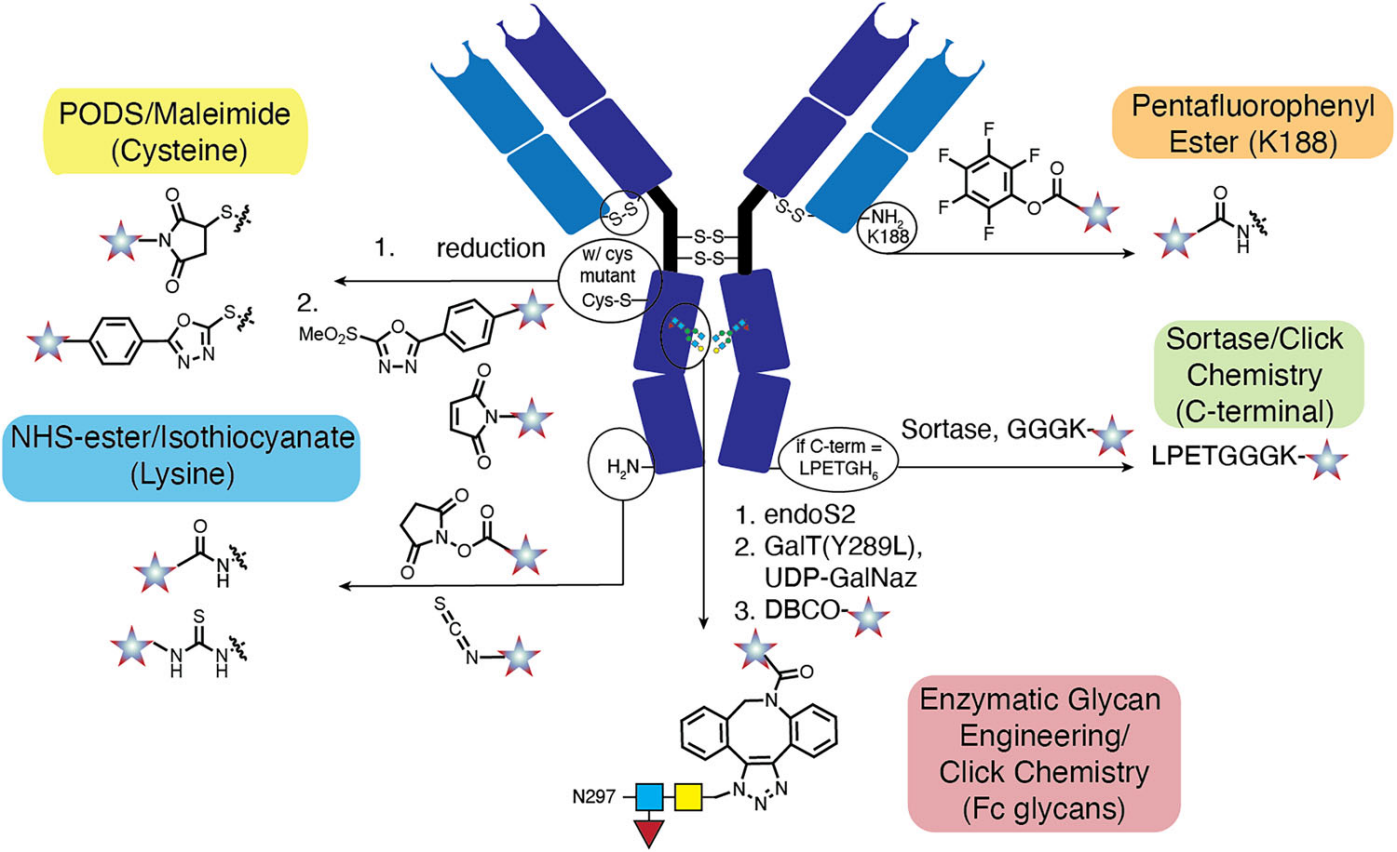
Bioconjugation chemistry. Bioconjugation chemistry and site of modification described in this review.

A critical consideration is bioconjugation chemistry. Conventional labeling of exposed lysines, typically with *N-*hydroxysuccinimide (NHS) esters or isothiocyanates, and interchain disulfide-linked cysteines, typically with maleimides, remain the most common approaches. While these approaches provide reliable access on large scale to modified mAbs, they come with inherent disadvantages. For lysine labeling, between 35-40 exposed sites (about half the total number) are typically modified following NHS ester modification in a stochastic manner^13^. The density of labeling can have a profound impact on mAb targeting, and the optimal number of modified lysines being typically less than 5 though this varies with the modification chemistry^14,15^. With respect to interchain cysteine labeling, the optimal payloads can stoichiometrically modify all 8 sulfur atoms leading to a relatively homogeneous product, though often lower labeling density is used^16^. However, first generation maleimides are prone to a reversible, retro-Michael reaction that liberates a fraction of the imaging probe leading to non-specific, untargeted uptake^17^. To address this issue, there are a variety of strategies using modified maleimides and other electrophiles that provide conjugates with improved stability^17–20^. In addition, many approaches have been developed that provide homogeneous products using a myriad of alternative chemistries, which have been reviewed elsewhere^21–24^. Below, we summarize examples where several of these strategies (Fig. 2) have been applied in paired imaging studies that allow for the impact of labeling chemistry to be clearly assessed.

To address the issue of maleimide stability, Barbas and coworkers reported that phenyloxadiazolesulfone (PODS) electrophiles react readily with cysteines to form stable conjugates^25^. The utility of this electrophile for [^89^Zr]Zr-deferoxamine (DFO) PET imaging was assessed by comparison to the maleimide derivative by Zeglis and coworkers^26^. After mAb labeling, the two conjugates were first compared for in vitro serum stability with the PODS derivative found to exhibit ~20% improved stability. Both conjugates were then appended at similar drug-to-antibody ratio (DAR, ~2) to an mAb targeting A33, a transmembrane glycoprotein frequently overexpressed in colorectal cancer. In vivo imaging in a colon cancer model (SW1222) found that while the two agents exhibit similar tumor targeting (50-60% ID/g), the signal from the PODS conjugation was reduced in the liver, kidney, spleen and bone. These changes result in a 1.8-fold improvement in the tumor to liver ratio (TLR) and a 1.6-fold improvement in the tumor to kidney ratio (TKR). These studies suggest that PODS conjugation is a viable option for cysteine bioconjugation at mAb sites prone to maleimide deconjugation, including the commonly used interchain disulfides.

A powerful strategy for homogeneous labeling is glycoengineering, which uses enzymatic means to modify the glycans natively found in the Fc region of mAbs. The potential for site-specific labeling for ^89^Zr-mediated PET imaging has been investigated extensively through efforts by Zeglis and Lewis^23,27^. These clinical efforts over the past two decades applying ^89^Zr-labeled HER2-targeting PET tracers have demonstrated the potential of these agents for applications in tumor staging, identifying HER2-positive tumors, and evaluating HER2 heterogeneity^2^. These studies, which used lysine-labeled DFO derivatives, also highlighted key limitations, including low uptake in some HER2-positive metastases and high background uptake in the liver and bone tissues that leads to reduced sensitivity^28^. To address these limitations, glycoengineering approaches were applied. These methods were originally developed for ADC purposes^29^, and rely on a pair of enzymes, EndoS and GalT(Y289L) to incorporate azide-containing sugars into the heavy-chain glycans in the Fc region of mAbs. This strategy then uses a strain-promoted azide-alkyne cycloaddition click reaction to attach the chelators to the enzymatically installed azido sugars leading to well-defined, homogeneous radioimmunoconjugates. Preclinical imaging efforts in models of HER2-positive breast cancer found that the homogeneous ^89^Zr-pertuzumab exhibited excellent in vivo behavior with improved performance relative to that of the stochastically lysine (isothiocyanate) labeled variant at similar labeling density. In a comparison study, the two agents were analyzed in a triple-negative breast cancer model (BT-474) leading to a 2.4-fold improvement in tumor signal and a 2.2-fold reduced liver and spleen signal (120 h post injection (p.i.)) for the homogeneous variant^30^. A recent clinical trial included a head-to-head comparison of both agents in a single patient with initial evidence suggesting the homogeneous variant was able to improve lesion detection and tracer targeting^28^. Overall, these studies suggest that homogeneous labeling through glycan modification can provide meaningful improvements in the performance of the PET imaging probe.

Nanobodies offer unique properties for imaging applications, including the potential for high-contrast early time point imaging. These targeting agents have been frequently modified with enzymatic labeling using the sortase enzyme, which cleaves a peptide sequence (LPXTG) to form a reactive thioester that reacts with an N-terminal glycine to form a new amide bond^31^. This approach was applied with a nanobody (VHH) antibody fragment against glypican-3 (GPC3), which is a glycosylphosphatidylinositol-anchored heparan sulfate proteoglycan expressed in >75% of hepatocellular carcinoma (HCC)^32^. Both the site-specific and heterogeneous variants at similar labeling displayed similar nanomolar affinity for GPC3 in vitro. Ex vivo biodistribution and PET/CT image analysis in mice bearing GPC3^+^ xenografts (A431 cells) found the homogeneous variant to have improved targeting, with a significant 2.3-fold improvement in the tumor-to-liver ratio (TLR), which suggests this agent may have utility as a liver cancer diagnostic. Other approaches have also been applied to the generation of homogeneous nanobody-based radioconjugates. For example, Massa and coworkers compared cysteine-tagged VHH to stochastic lysine-based conjugation and found comparable performance^33^. Another issue that has also been investigated is the impact of common C-terminal tags, namely MYC and His, on the biodistribution of a HER2-targeted VHH^34,35^. These studies found each tag significantly increased renal retention indicating that, while useful for purification and characterization, they should be removed prior to translation.

Labeling chemistry has also been examined in the context of fluorophore-labeled mAbs. Immunolabeling approaches are extensively used for microscopy, flow cytometry, and ex vivo imaging of fixed tissues. Extending these approaches to in vivo imaging for guided surgical applications was driven by extensive preclinical and clinical efforts by Rosenthal and coworkers, and first applied clinically with conjugates of IRDye-800CW and cetuximab in head and neck cancer^36^. While largely unaffected by low label density (Degree of Labeling - DOL < 1), at even moderate probe density (DOL 2-3)^15,37^, IRDye-800CW conjugates show significant hepatic signal and only modest tumor contrast. Efforts to address this have included altering the bioconjugation chemistry, described in this section, and changing the labeling agent, which is described in the subsequent section.

**Fig. 3:**
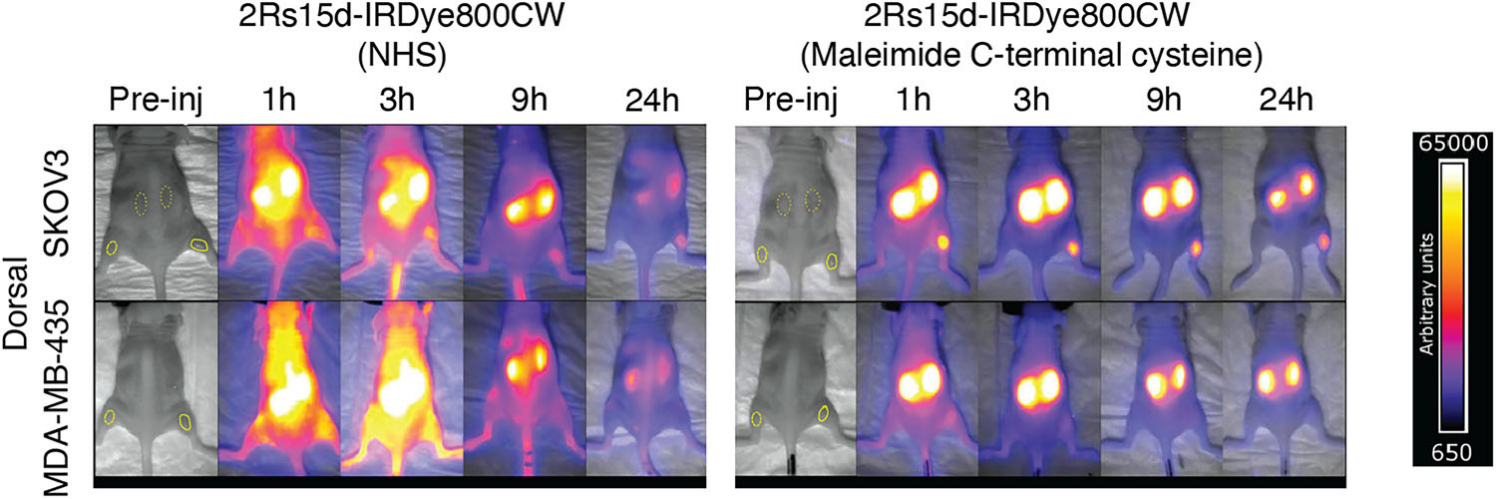
Impact of homogeneous labeling on nanobody imaging. 2D fluorescent images (both dorsal views) of biodistribution and tumor targeting of randomly (right) and site-specifically (left) IRDye800CW-labeled HER-2 targeting nanobody in SKOV3 (HER2+) and MDA-MB-435 (HER2–) xenografts. Figure adapted from ref. ^39^

Site-specific labeling using engineered cysteines, termed “thiomab labeling”, was first applied by Genentech for ADC development^38^. This approach was applied for optical imaging by Hernot and coworkers for targeted nanobodies labeled with IRDye-800CW (Fig. 3)^39^. The site-specific labeled IRDye-800CW nanobody conjugate showed rapid tumor accumulation with useful tumor-to-background ratio (TBR), while the stochastic lysine labeled conjugates showed higher liver and other off-target uptake and delayed selective tumor accumulation. Significant differences persist to 24 h, with a 4.1-fold difference in the tumor-to-muscle ratio (TMR). Another example of this approach was reported by St. Croix in the context of a full-length mAb targeting CD276 (B7-H3)—an emerging target expressed in a range of solid tumors. In this study, a homogeneous doubly labeled at a S239C mutant cysteine position was compared to a hinge labeled maleimide of similar DOL using a relatively hydrophobic heptamethine cyanine derivative. When imaged in a triple-negative breast cancer model (JIMT-1), the homogeneous variant showed 4.2-fold improved tumor-to-background ratio (TBR) and 3.1-fold reduced liver signal compared to the heterogenous variant. These results suggest that embedding the hydrophobic cyanine within the Fc domain adjacent to the glycan may limit the exposure of the probe to interacting proteins, especially when compared to the relatively solvent-exposed interchain cysteine sites^40^. This study also details extensive characterization of the S239C pyrrolobenzodiazepine ADC, which exhibits potent antitumor activity in a range of preclinical models.

Lastly, we describe an approach to site-specific labeling that uniquely targets a native lysine on human mAbs without requiring genetic or enzymatic manipulation. This approach, also originally described for ADC applications, involves the use of pentafluorophenyl (PFP) esters and low temperature (4 °C) conditions to engage a uniquely reactive lysine (K188) found in the human kappa light chain^41^. This method was applied for optical imaging applications by Schnermann and coworkers comparing the in vivo targeting of a hydrophobic cyanine label on panitumumab, appended with either high light-chain specificity using a PFP-derivative or non-selectively using conventional NHS-ester conditions^42^. When imaged in a triple-negative breast cancer (MDA-MB-468) xenograft model, the PFP derivatization strategy improved in vivo targeting relative to a non-selective NHS-ester conjugates of similar DOL with a 1.9-fold improvement in TBR. Zeglis and coworkers applied this approach to ^89^Zr-PET imaging through the use of a branched bisazide-bearing PFP ester, followed by click chemistry attachment of dibenzocyclooctyne (DBCO)-bearing DFO derivatives^43^. The resulting high purity conjugates exhibit improved in vitro binding, but no statistically significant improvement in in vivo tumor targeting in the BT-474 HER2+ breast cancer model, when compared to maleimide or NHS-ester conjugates. The PFP strategy provides a uniquely simple to implement approach for homogeneous mAb modification without reliance on either enzymatic modification or protein engineering. Overall, these labeling strategies provide homogeneous imaging probes with improved in vivo properties. We note that while we have highlighted examples where homogeneous labeling chemistry improved imaging, there are also examples where conventional labeling yields conjugates with similar or even improved in vivo performance^44,45^. Additionally, the more common heterogeneous labeling methods benefit from well-established work-flows and ready scalability. Therefore, future work will likely center on establishing settings where homogeneous chemistries provide clinically meaningful benefit.

## Imaging agent

The chemical composition of the imaging agent also plays a central role in the properties of the protein conjugate. Critically, the demands on the chemical properties of the probe vary significantly between optical and radionuclide-based imaging. For radiometal-based conjugates, the challenge is two-fold: first, to ensure covalent linkage of chelator and protein, and second, to maintain a stable chelate-radioisotope complex and entire radioconjugate, both in circulation and following target engagement in situ. In contrast, the challenge in optical imaging is to identify bright, stable fluorophores with emission properties shifted into the longer wavelength range, where absorption by endogenous chromophores is minimized^46,47^. In this regard, heptamethine cyanines have significant advantages for targeted imaging, including excellent optical properties in the near-infrared (NIR) range and significant preclinical and clinical infrastructure using the relevant wavelengths^48^. A critical issue is converting the intrinsically high molecular weight, hydrophobic cyanine scaffold into a hydrophilic agent that minimally alters the properties of the parent antibody^49^. Below, we highlight the state of the art in both areas with examples that systematically looked at the consequences of varying the imaging probe component.

**Fig. 4:**
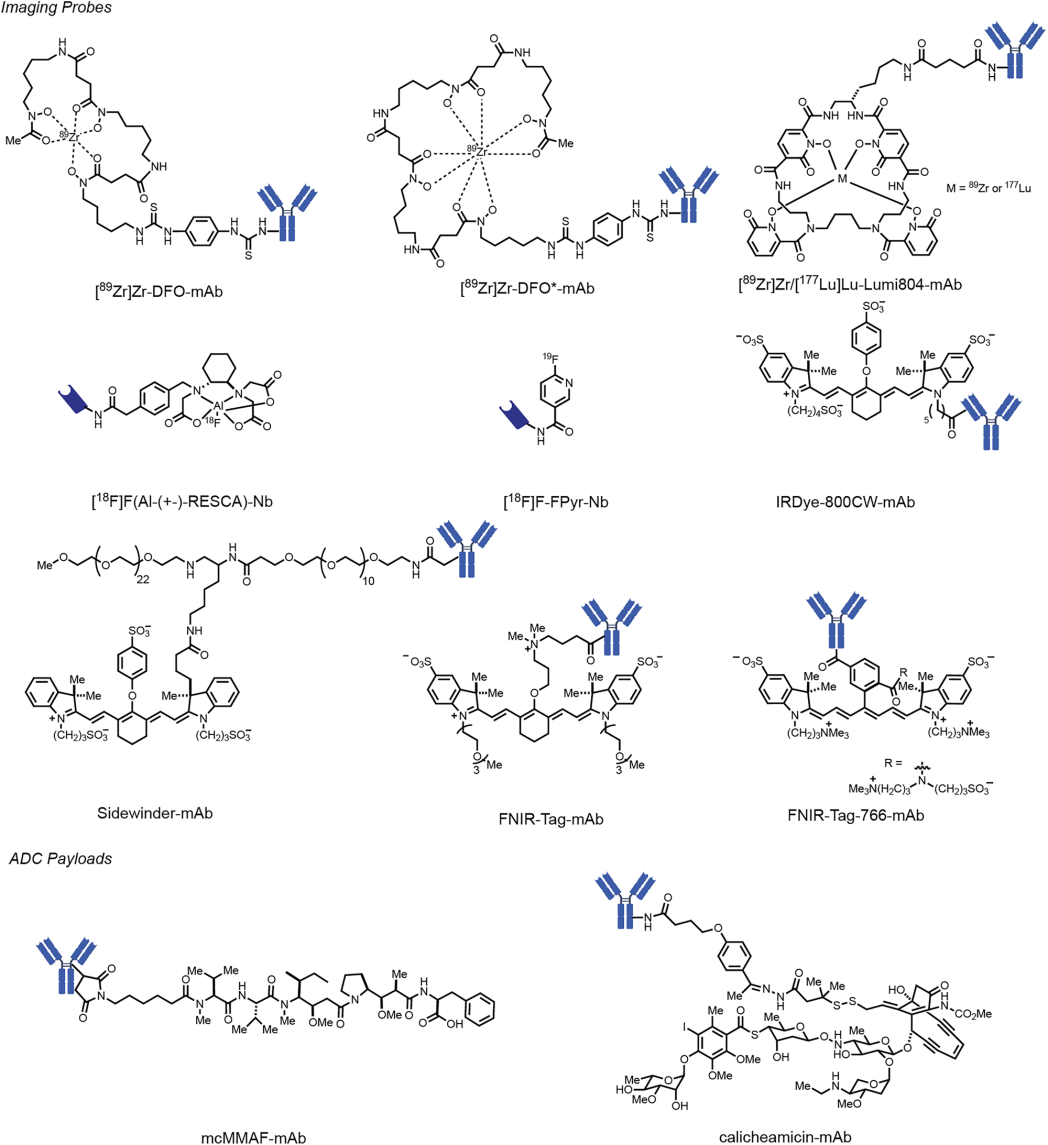
Chemical structures. Structures of imaging probes and ADC payloads described here.

A common choice for antibody radionuclide imaging is the polyhydroxamic acid DFO, which is a naturally occurring chelator first known for its ability to bind iron. The ability of DFO to form complexes with a range of radiometals including ^68^Ga, ^89^Zr, and ^111^In, has been harnessed through the creation of variants that allow for protein conjugation. While broadly used, the modest stability of DFO conjugates leads to significant loss of the chelated species, resulting in off-target signal dependent on the identity of the imaging radionuclide and its tropism for certain tissues (e.g., free ^89^Zr accumulates in bone). DFO* was developed to overcome limitations of the original DFO by providing a complete coordination sphere through an additional hydroxamic acid (Fig. 4)^50^. Vugts and coworkers compared the two chelators for ^89^Zr-immuno-PET imaging of trastuzumab conjugates in a N87 xenograft murine model^51^. These studies found that [^89^Zr]Zr-DFO*-trastuzumab exhibited improved in vitro serum stability compared to [^89^Zr]Zr -DFO-trastuzumab. As a consequence, in vivo studies showed that, while the agents maintained comparable tumor targeting, [^89^Zr]Zr-DFO*-trastuzumab exhibited significantly lower bone uptake (5.9-fold) and reduced uptake in skin, liver, spleen, and ileum. These properties make DFO* a promising candidate to replace DFO in clinical ^89^Zr-based immuno-PET applications. Lumi804 is a novel chelator that can form complexes with a variety of radionuclides, including ^89^Zr and ^177^Lu^52^. When [^89^Zr]Zr-DFO-αCD11b and [^89^Zr]Zr-Lumi804-αCD11b were compared, the DFO variant displayed higher bone signal (13-fold), due to accumulation of released free ^89^Zr, along with 5.2-fold higher kidney and 24-fold higher intestinal accumulation. Overall, while there are significant limitations of DFO chelates, several useful alternatives are available and being actively explored.

The issue of chelator chemistry and site-specific labeling was recently addressed for early time point antibody fragment imaging in a study by Jacobsen and coworkers^53^. This study investigated ^18^F-labeled variants of an EGFR-targeting nanobody, which was varied with respect to both the chelator/prosthetic group and the nature of the linkage chemistry. The study compared nanobodies modified with a constrained tricarboxy diamine (i.e., a restrained complexing agent, or RESCA)-aluminum-fluoride (Al[^18^F]F) complex (Fig. 4) and a 2-fluoropyridine derivative, [^18^F]-FPy in an A431 squamous cell carcinoma model. Using heterogeneous lysine labeling, the higher molecular weight Al[^18^F]F showed high kidney retention, which could be partially reduced through PEGylation, though this decreased tumor uptake. By contrast, the [^18^F]F-FPy prosthetic groups exhibit much more favorable properties, showing rapid renal clearance and minimal kidney retention while maintaining higher tumor uptake (~4-fold), leading to a vastly improved tumor-to-kidney ratio (TKR) (>30 vs <1 for the Al[^18^F]F conjugates). Of note, others have previously reported that radiometal-based conjugates tend to exhibit higher kidney uptake when compared to non-metal-based radioconjugates^35,54^. This study also assessed the impact of sortase conjugation on this approach and showed only small, almost negligible effects, when carefully comparing RESCA complex labeling strategies. These findings suggest the potential for using pyridine derivatives for clinical translation of diagnostic agents. Furthermore, similar approaches to minimize the kidney absorbed dose could enable production of targeted radiopharmaceutical therapy agents using radioisotopes like ^131^I or ^211^At.

Several strategies have sought to improve the imaging properties of mAb-fluorophore conjugates. The most common chemical strategy to improve fluorophore properties for biomolecule labeling is to install multiple sulfonate groups around the periphery of the probe. This approach forms the basis of the commonly used “Cy” dyes and Alexa Fluor series^55^. IRDye-800CW follows in this tradition being developed by the LICOR group for DNA- and protein-labeling applications. As mentioned above, while suitable for in vitro purposes, persulfonation results in high off-target, typically hepatic, uptake in live animal imaging. There have been several recent studies seeking to address the limitations of IRDye-800CW conjugates through chemical modifications. One example is the sidewinder probe, which is a PEGylated variant of IRDye-800CW that was tested in the context of an anti-CEA mAb in a murine model of pancreatic cancer. These compounds were used as bromoacetamides, an alternative electrophile for interchain cysteine labeling, and compared to conventional IRDye-800CW NHS ester lysine conjugates. The sidewinder strategy resulted in increased tumor fluorescence and a higher tumor-to-background ratio (TBR) (1.8-fold) in a colorectal cancer mouse model (LS174T). This work suggests that well-placed PEG groups can improve the properties of imaging agents^56^.

**Fig. 5:**
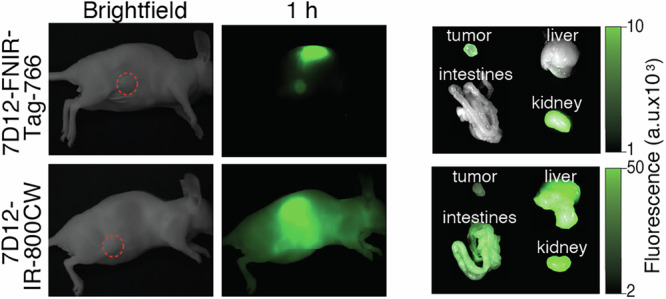
Impact of fluorescent probe on nanobody imaging. In vivo fluorescence images (left) of orthotopic JIMT-1 tumor bearing mice intravenously injected with 7D12-FNIR-Tag-766 and 7D12-IRDye-800CW (DOL 1.1, 2 nmol) at 1 h postinjection. Tumors are highlighted in red dotted circles. Ex vivo biodistribution (right) of 7D12-dye conjugates on orthotopic JIMT-1 tumor-bearing mice after 1 h post-injection. Figure adapted from ref. ^61^.

Another strategy is to alter the charged functional groups that are appended to the core chromophore unit. Studies by Choi, Gibbs, and Frangioni reported that heptamethine cyanine modified with a combination a indolenine trimethylpropyl ammonium and aryl sulfonate substituents improved the in vivo targeting of peptide-based imaging agents^57^. Notably, one agent to emerge from these efforts, ZW800, as a cyclic-RGD conjugate has advanced to clinical testing^58^. Efforts to optimize mAb fluorescent labels for tumor imaging were carried out in iterative efforts by the Schnermann group^59–62^. Using a synthetic method that provides access to C4′-O-alkyl heptamethine cyanines, a series of sulfonated and zwitterionic cyanines were prepared and tested. These studies lead to the first generation Frederick-NIR (FNIR)-Tag, which, contains charged groups both on the periphery and, unlike previous agents, adjacent to the polymethine chromophore^63^. This agent and related agents improve imaging properties across various types of targeting agents, including peptides, nanoparticles and mAbs. For example, FNIR-Tag was compared to IRDye-800CW, using NHS-ester mediated lysine modification using panitumumab in an MDA-MB-468 model. This compound improved the tumor-to-background ratio (TBR) (by 3.9-fold) and decreased the liver-to-background ratio (LBR) (by 4.6-fold). Further optimization has recently been enabled by cyanine preparative methods that start from pyridine precursors^64^. These studies demonstrated that C4′ aryl derivatives exhibit a unique combination of excellent optical properties and potential for further modification. Examples of arylated cyanines include FNIR-Tag-766 and s775z, another broadly used cyanine derivative^65^. FNIR-Tag-766 was applied for both intact mAb and nanobody based imaging^61^. When applied for the latter, this agent was compared to IRDye-800CW lysine conjugates, where FNIR-Tag-766 was found to dramatically improve tumor-to-liver ratios (TLR) (by 12.5-fold) at early 1 h time points in a JIMT-1 model (Fig. 5). The key molecular design insight is the role of charged functional groups or steric bulk directly adjacent to the chromophore unit. This chemical strategy serves to block the formation of undesirable dye-dye interactions while likely reducing certain biomolecule interactions. More broadly, these studies highlight the important role of probe chemistry optimization in the development of in vivo imaging strategies.

## Antibody drug conjugates

Antibody-drug conjugates (ADCs) are designed to specifically deliver drug payloads to target-expressing cancer cells, while minimizing exposure to healthy tissue^66^. In practice, a large fraction of the ADC payload is not delivered to the tumor site, which can limit the therapeutic effectiveness of this strategy^67^. Monitoring the distribution of both the mAb and the free drug throughout tissues is essential for gaining a comprehensive understanding of ADC properties. Measuring mAb blood levels using ELISA-based measurements is common but assesses changes due to drug conjugations in an indirect manner (i.e., changes relative to the parent antibody). Approaches to examine the payload directly in tissue include isotopic labeling studies with ^14^C or ^3^H, followed by ex vivo scintillation counting, and high-resolution mass spectrometry^68^. PET/SPECT and optical imaging strategies enable real-time noninvasive monitoring of payload distribution in live animals, either on intact ADC or free drug following cleavage. Below we summarize several studies in which the impact of a drug payload-mAb modification was assessed quantitatively using in vivo imaging.

Several studies have used optical methods to compare the distribution of ADCs and the corresponding parental mAb. The impact of the conversion of daratumumab (DARA), an FDA-approved anti-CD38 monoclonal antibody for multiple myeloma, into an antibody-drug conjugate was investigated by Shokeen and coworkers^69^. This study compared DARA modified with the fluorescent dye IRDye800-CW (DARA-IR) to a version conjugated to the microtubule-targeting maytansine derivative DM1 and the same dye (DARA-DM1-IR). As expected, the ADC improves therapeutic efficacy compared to the unmodified antibody. However, in vivo fluorescent imaging showed that the liver uptake of DARA-DM1-IR was 1.8-fold higher than DARA-IR and the tumor uptake was slightly reduced (1.2-fold) in a multiple myeloma model (MM.1S). Giddabasappa and colleagues evaluated the biodistribution and tumor targeting of an anti-5T4 antibody and a 5T4-ADC conjugated to the auristatin derivative MMAF^70^. 5T4, or trophoblast glycoprotein, is a heavily N-glycosylated protein associated with the directional movement of cells through epithelial mesenchymal transition and is associated with a variety of solid tumors. The study sought to assess whether maleimide conjugation of MMAF affected biodistribution in an H1975 non-small cell lung cancer subcutaneous xenograft model using fluorescence molecular tomography (FMT) imaging. To track distribution, they attached the fluorescent tag VivoTag 680XL (VT680) to both the antibody and its ADC using conventional NHS lysine labeling. In vitro, the labeled 5T4-Ab and 5T4-ADC showed similar stability and binding affinity. However, in vivo imaging demonstrated that 5T4-ADC-VT680 exhibited a 1.5-fold reduction in tumor accumulation compared to 5T4-Ab-VT680 at 10 days post-injection with significant hepatic signal for both agents. The common theme to emerge from these efforts is a deleterious effect of drug conjugation on tumor targeting, which is likely at least partially attributable to enhanced hepatic clearance by the reticuloendothelial system.

A study conducted by Jacobson and colleagues used PET imaging to guide the evaluation of anti-EphrinA (EphA2) antibodies and their drug conjugates. The authors investigated how varied internalization rates and hydrophobicity influence tumor delivery with and without drug conjugation. The antibodies, 1C1, 3B10, and 2H7, were selected through a phage-based approach with binding constants all in the low nanomolar range. In addition, their respective ADCs were created through a site-specific S239C mutation conjugation, resulting in homogeneous ADCs with a drug-to-antibody ratio (DAR) of 2 after conjugation with the tubulysin payload AZ13599185. Using ^89^Zr-based PET imaging in a PC-3 prostate cancer model, investigators found a strong correlation between hydrophobicity and in vivo tumor uptake, with the least hydrophobic antibody, 3B10, showing the highest tumor uptake. In vivo PET imaging comparing ^89^Zr-labeled mAbs and ^89^Zr-labeled ADC found that the ADCs exhibited 1.9-fold lower tumor uptake across the series^71^. These studies provide an additional example of the impact of drug modification on tumor targeting.

A final example of the impact of drug modification on mAb targeting is a clinical study by Herbertson and coworkers^72^. This Phase I study investigated the biodistribution and pharmacokinetics of the immunoconjugate CMD-193, which consists of hu3S193, a humanized monoclonal antibody specific to the difucosylated tetrasaccharide Lewis Y antigen that is overexpressed in several epithelial cancers, conjugated with cytotoxic calicheamicin via non-selective lysine labeling. Both hu3S193 and its ADC derivative were modified with CHX-A-DTPA, a pentacarboxy triamine using isothiocyanate lysine labeling and imaged longitudinally with ^111^In-SPECT imaging. The study found that [^111^In]In-CMD-193 exhibited more rapid blood clearance, increased hepatic uptake (by 4.5-fold), and dramatically reduced tumor uptake (by 19-fold) compared to the labeled parental antibody [^111^In]In-hu3S193. This study suggests that impacts of mAb labeling seen in murine studies can translate to human settings. Understanding the biodistribution of the parental antibody, the ADC, and free drug informs both the potential efficacy and toxicity of the ADC, suggesting such studies should be incorporated early in the ADC development process.

## Alternative strategies

Finally, we review several alternative strategies to improve mAb targeting that have been recently assessed using quantitative paired in vivo imaging studies. The long serum half-lives of mAbs are due in large part to neonatal Fc receptor (FcRn)-mediated recycling. However, this property comes with a requirement of a long interval (days) between injection and imaging to achieve high tumor contrast with full-length antibody-based imaging agents. To address this challenge, Fc-mutations that reduce interaction with the FcRn receptor have been identified and incorporated into various targeting mAbs. A study investigating this approach was recently reported by a Genentech group with engineered antibodies that modify FcRn binding^73^. The study explored how FcRn-mediated interactions influenced serum and tumor-to-background ratios (TBR) through mutations: H310A/H435Q (HAHQ) to block FcRn binding in a HER2-binding antibody. The study used a breast cancer xenograft mouse model (KPL-4) and found that these mutations indeed reduce blood level (by 4.2-fold) but also reduce modestly tumor levels (by 1.7-fold) 48 h post injection. Notably, a recent study found that an alternative Fc mutation (LALAPG), which reduces mAb interaction with Fcγ receptors without substantial changes in vivo tumor targeting^40^. These studies suggest that, while Fc mutations may alter tumor uptake, such strategies can accelerate blood clearance and have significant potential to address certain target or disease specific challenges. We note that mAb isotype can also play a profound role on in vivo pharmacokinetics, which has been reviewed extensively elsewhere^74–76^.

**Fig. 6:**
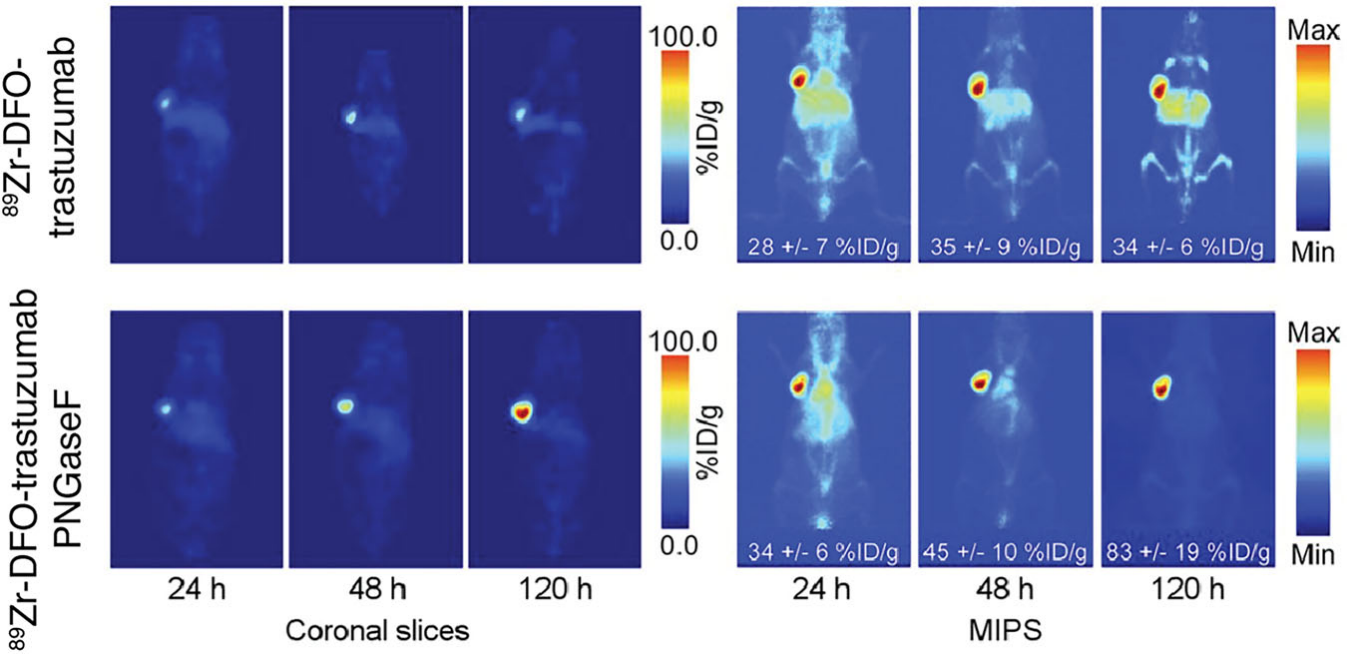
Impact of Fc deglycosylation on mAb imaging. In vivo imaging of [^89^Zr]Zr-DFO-trastuzumab and [^89^Zr]Zr-DFO-trastuzumab-PNGaseF. Planar (left) and maximum-intensity projection (right) PET images of humanized NSG mice bearing subcutaneous BT474 xenografts at 24, 48, and 120 h after injection. Figure adapted from ref. ^78^.

In addition to the primary sequence of the Fc domain, the glycan attached at Asn297 plays a key role in the recognition by various Fc receptors (particularly the FcγRI receptor)^77^. Zeglis and coworkers explored wheather the impact complete removal of antibody glycosylation could improve PET imaging performance by reducing unwanted uptake mediated by FcγRI (Fig. 6)^78^. Using the common mAb deglycosylation enzyme, Peptide:N-Glycosidase F (PNGase F), they generated deglycosylated trastuzumab, which was compared to the parent glycosylated variant following non-selective desferrioxamine conjugation and radiolabeling with ^89^Zr. As anticipated, full deglycosylation significantly reduced biochemical binding to the human FcγRI receptor. Notably, partial deglycosylation with EndoS and modification with a DFO chelator also moderately reduced binding, but to a less degree than complete deglycosylation. Several of these agents were then examined in murine imaging studies. The magnitude of the impact of deglycosylation was found to depend heavily on the mouse models employed. However, imaging studies in a humanized model (irradiated NSG mice reconstituted with hematopoietic stem cells) bearing a BT-474 xenograft, found that the deglycosylated variant exhibited 2.1-fold lower liver and 3-fold lower spleen uptake (Fig. 6). These findings suggest that antibody deglycosylation represents a straightforward approach to enhance the quality of immuno-PET imaging.

Several approaches have sought to reduce off-target exposure of radioconjugates and ADCs due to long circulating half-lives of intact mAbs. One example is pretargeting that uses an initial antigen targeting agent, which is given time to accumulate at the tumor site, followed by addition of imaging/therapeutic ligand or click chemistry reactant that can bind/react with the targeting agent and exhibits rapid clearance^79^. Early pretargeting pairs included streptavidin/biotin and oligonucleotides^80^. More recent efforts have benefited from the development of extremely rapid click reactions, specifically those between tetrazine and trans-cyclooctenes^81,82^. A study quantifying the in vivo tumor targeting potential of this approach when compared to conventional mAb targeting was reported by a Genentech group. The HER2-targeting antibodies were tested using both direct (^111^In-DOTA) and pretargeted imaging using in vivo click chemistry between trans-cyclooctene (on the mAb) and tetrazine (on DOTA). Using KPL-4 tumor bearing mice, investigators found that direct labeling resulted in 4.7-fold higher tumor uptake and 2-fold higher tumor-to-blood ratios compared to pretargeting at 48 h^73^. Additionally, although the goal of pretargeting is to reduce off-target uptake in excretory organs such as the liver and spleen, the direct mAb targeting approach exhibited lower uptake in these organs compared to those of the pretargeting approach. These findings suggest that significant optimization is still needed to realize the goals of click-based pretargeting strategies.

Predosing or codosing with an unconjugated mAb is a strategy that can reduce ADC uptake in non-tumor tissues, while still allowing for effective tumor targeting. This approach has been studied in various ways, including by using the parent unlabeled antibody in combination with the modified therapeutic agent^83^. A recent study explored the potential of pre-dosing using anti-TENB2 mAbs and the corresponding ADCs with the goal of enhancing ADC safety for prostate cancer treatment^84^. Administration of ^111^In-DOTA-anti-TENB2-MMAE antibody was assessed in isolation or following predosing of the anti-TENB2 mAb at doses of 1 mg/kg, 3 mg/kg and 10 mg/kg in LuCaP 77 prostate cancer model. The 1 mg/kg predose maintained high tumor uptake similar to that seen without predosing while leading to a reduction in liver signal (by 2.1-fold) and intestinal signal. However, at the 3 mg/kg or 10 mg/kg doses tumor signal was dramatically reduced (by 3-fold and 10-fold, respectively) indicating a dose-dependent and antigen-specific effect (e.g. blocking). While this study suggests that co-dosing can reduce off-target uptake, careful, and, perhaps, patient-specific dosing may be required.

## Conclusion

In vivo imaging has the potential to provide quantitative insights into the fate of labeled therapeutic proteins in living systems. Here, we have shared some studies describing the degree to which in vivo antibody biodistribution is impacted by chemical modifications. Several homogeneous labeling chemistries are available to yield chemically pure bioconjugates. While the impact of such methods appears to be context specific, there are many benefits of generating a single well-defined molecular species and these methods will continue to be applied in many settings. In addition to labeling chemistry, the chemical composition of the imaging agents also plays a critical role on in vivo distribution. Recent efforts have identified new chelators with improved in vivo stability and fluorescent probes with altered charged distribution and greater hydrophilicity. These new imaging agents significantly improve in vivo tumor targeting, while reducing the off-target uptake, and have significant potential for future translation. Critically, the origin of these effects is multifactorial, arising from likely combination of changes in target affinities, conjugate stability and, perhaps most significantly, altered interactions with clearance pathways. It is also critical to note that in vivo imaging assesses only the final disposition of the imaging probe itself at a whole-body resolution. Therefore, it must be used in combination with other techniques, such as biochemical affinity measurements and pharmacokinetic parameter assessments, to fill in key details.

A critical theme in this review is the important role of imaging in the characterization of new antibody-based therapeutics. Radiotheranostics have long benefited from the direct conversion between imaging probes and their therapeutic derivatives—an approach that is now firmly established and reviewed elsewhere^85^. The development of ADCs can also benefit from both radionuclide-based and optical imaging “lenses”. Radionuclide-based imaging provides highly quantitative assessment of in vivo biodistribution, allowing for early identification of off-target accumulation of either the ADC or the free drug that may predict toxicity. Optical probe-based strategies can also assess in vivo distribution, while offering the additional benefit of providing cellular level resolution of tumor penetration. Detailed studies by Thurber and others have demonstrated the significant potential for studies that span in vivo to ex vivo cellular imaging, including in recent clinical studies^8,67,86^. Furthermore, efforts that combine ADCs with therapeutic isotopes are an emerging area with potential for further exploration, however the impact of extensive antibody modification on biodistribution should be carefully considered^87^. In addition, efforts by our group and others are leading to mAb imaging probes that are activated by pH or proteolytic cleavage allowing for quantitative assessment of mAb internalization and linker cleavage^15,45,88^. In aggregate, these approaches provide highly quantitative insights into the chemical fate of therapeutic proteins and, critically, have the potential to assess these issues early in the ADC development process.

Finally, there are several methods that can be used to improve biodistribution and pharmacokinetics of mAb-based agents, including, pretargeting, Fc modifications, and pre- or co-dosing with parental antibody. Alternative labeling chemistries and targeting agents also present opportunities to tune the in vivo performance of such agents^89,90^. Which of the aforementioned strategies is selected may depend on the desired performance of a given agent. As novel agents (e.g. bispecific T-cell engagers, cell therapies, other biomolecule-drug conjugates) emerge, employing early quantitative in vivo imaging can provide critical early data on their on-tumor and off-tumor distribution informing on issues of both efficacy and toxicity^91–93^.

## Data Availability

No datasets were generated or analyzed during the current study.
